# L-Arginine-Derived Polyamidoamine Oligomers Bearing at Both Ends β-Cyclodextrin Units as pH-Sensitive Curcumin Carriers

**DOI:** 10.3390/polym14153193

**Published:** 2022-08-05

**Authors:** Sofia Treccani, Jenny Alongi, Amedea Manfredi, Paolo Ferruti, Roberta Cavalli, Giuseppina Raffaini, Elisabetta Ranucci

**Affiliations:** 1 Dipartimento di Chimica, Università degli Studi di Milano, Via C. Golgi 19, 20133 Milano, Italy; 2 Dipartimento di Scienza e Tecnologia del Farmaco, Università degli Studi di Torino, Via P. Giuria 9, 10125 Torino, Italy; 3 Dipartimento di Chimica, Materiali, e Ingegneria Chimica “Giulio Natta”, Politecnico di Milano, Piazza L. Da Vinci 32, 20131 Milano, Italy

**Keywords:** β-cyclodextrin, polyamidoamines, curcumin, drug release, molecular modeling

## Abstract

The aza-Michael polyaddition of L-arginine and *N*,*N*′-methylene-bis-acrylamide gives the biocompatible and easily cell-internalized polyamidoamine ARGO7. By controlled synthesis, two ARGO7 oligomers, namely a trimer and a pentamer, bearing acrylamide terminal units, were obtained as precursors of the β-cyclodextrin-end-terminated oligomers P3 and P5, which have been shown to encapsulate curcumin at both pH 7.4 and 4.5. After lyophilization, P3- and P5-curcumin complexes gave stable water solutions. The apparent solubility of encapsulated curcumin was in the range 20–51 μg mL^−1^, that is, three orders of magnitude higher than the water solubility of free curcumin (0.011 μg mL^−1^). The drug release profiles showed induction periods both at pH levels 4.5 and 7.4, suggesting a diffusive release mechanism, as confirmed by kinetic studies. The release rate of curcumin was higher at pH 7.4 than at pH 4.5 and, in both cases, it was higher for the P5 complex. Encapsulated curcumin was more photostable than the free drug. Molecular mechanics and molecular dynamics simulations explain at atomistic level the formation of aggregates due to favorable van der Waals interactions. The drug molecules interact with the external surface of carriers or form inclusion complexes with the β-cyclodextrin cavities. The aggregate stability is higher at pH 4.5.

## 1. Introduction

Cyclodextrins (CDs) are toroidal-shaped macrocycles containing six (α-CDs), seven (β-CDs) or eight (γ-CDs) α-glucopyranoside units [1]. CDs have a hydrophilic surface and a hydrophobic cavity and, with specific guest molecules, form inclusion complexes whose stability depends not only on the hydrophobicity of the guest molecules but also on their shape and size, which must match those of the internal CD cavity. This property is maintained when CD moieties are inserted in macromolecular compounds, in which case the different CD units can behave cooperatively [2]. This has also been exploited in CD nanosponges, which are crosslinked structures in which many CD units are covalently linked together forming nanochannels [3]. CD-containing polymers and nanosponges have received considerable attention in recent years [4,5,6,7].

CD complexes find relevant applications in many sectors [8], including food [4], pharmaceuticals [9,10], cosmetics, personal care [4], and in the environment [11]. A widespread application of CDs is in the pharmaceutical sector, for example, to improve the solubility, stability and bioavailability of hydrophobic drugs [9]. Curcumin, a natural yellow pigment extracted from the rhizomes of *Curcuma longa*, is used in a variety of pharmacological applications, including as an anticancer, antimutagenic, antibacterial, anti-inflammatory, antifungal, anti-allergy and antioxidant agent [12]. Free curcumin is very poorly soluble in water at acidic and neutral pH levels (0.011 μg mL^−1^ in pH 5 buffer solution) [13] and, consequently, poorly bioavailable. At basic pH levels it undergoes rapid degradation. Furthermore, it is characterized by limited thermal and oxidative stability and photostability [13]. Different nanoparticle formulations have been proposed to improve stability and bioavailability of curcumin, including polysaccharide-based formulations [14] and poly(2-vinyl pyridine)-b-poly(ethylene oxide) block copolymers [15], just to mention a few. In addition, curcumin forms inclusion complexes with CDs, greatly increasing solubility and stability [13,16,17].

In the last 20 years, CDs and their complexes have shown self-association processes in solution to form aggregates or micelle-like structures [18,19]. Furthermore, theoretical studies based on molecular mechanics (MM) and molecular dynamics (MD) simulations have proven to be powerful tools to explain at the atomistic level the stability and the conformation of different native or functionalized CDs, the geometry of host-guest inclusion complexes and β-CD nanosponges in good agreement with the NMR experimental data [20,21,22,23,24,25].

Polyamidoamines (PAAs) are multifunctional hydrophilic polymers obtained by the aza-Michael polyaddition of prim- or bis-sec-amines, including natural α-amino acids, with bis-acrylamides [26]. The reaction is usually carried out in water or protic solvents at room temperature, pH 8–10, and in the absence of added catalysts. Under these conditions, the reaction is highly selective since, in addition to the prim- or sec-amines, only the thiol and phosphine groups can give 1,4-conjugate addition with acrylamides. Many PAAs are biocompatible and, therefore, have been extensively studied for biotechnological applications [27,28]. In particular, the PAA-coded ARGO7 deriving from the polyaddition of L-arginine with *N*,*N*′-methylene-bis-acrylamide is highly water-soluble and biocompatible and, furthermore, susceptible to cell internalization [29]. ARGO7 oligomers with a controlled sequence of the monomeric units, controlled polymerization degrees, and predetermined terminal groups can be obtained by a step-wise polyaddition process [30]. In particular, acrylamide α,ω-end-capped ARGO7 oligomers susceptible to Michael-type addition reaction with thiols can easily be obtained that can be reacted with 6-deoxy-6-mercapto-β-cyclodextrin (β-CD-SH) giving β-cyclodextrin α,ω-end-functionalized amphoteric oligomers with predetermined distance of the two CD units. These are endowed with the potential to cooperate in forming water-soluble inclusion complexes with curcumin. The aim of this paper is to report on the results obtained in this field.

## 2. Materials and Methods

### 2.1. Materials

β-Cyclodextrin (β-CD, 95%) was purchased from abcr Gmbh (Karlsruhe, Germany); toluene-4-sulfonyl chloride (≥99%), potassium thioacetate (98%), sodium hydroxide (100%), acetonitrile (>99.9%), acetic acid (>99.8%), D_2_O (99.9 atom% D) and DMSO-d_6_ (99.96 atom% D), 2-propanol (>99.8%), L-arginine (Ar, ≥98.5%), *N*,*N*′-methylene-bis-acrylamide (M, 99%), 2,6-di-*tert*-butyl-*p*-cresol (98%) and hydrochloric acid (HCl, 37% *w*/*w*) were supplied by Sigma–Aldrich (Milano, Italy) and used as received. Lithium hydroxide monohydrate (≥98%), amberlite IRC-86 (H^+^-form; 20–50 mesh), *N*,*N*-dimethylformamide (DMF) (99.8%) were purchased from Fluka (Milano, Italy). Acetone ExpertQ^®^ ACS, ISO, Reag. Ph. Eur. was purchased from Scharlau (Barcelona, Spain).

De-oxygenated water and 2-propanol were obtained by purging nitrogen for 10 min.

### 2.2. Characterizations

**Figure 1:**
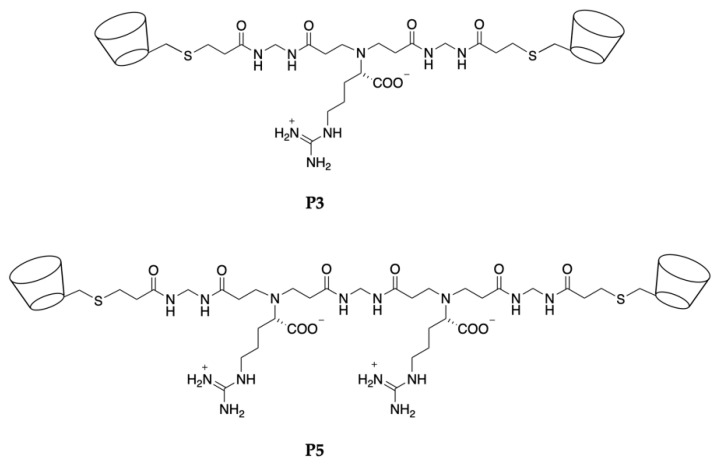
Structures of the P3 and P5 β-cyclodextrin α,ω-end-functionalized PAA oligomers.

The chemical structure of P3, P5 (Figure 1) and their precursors was assessed by ^1^H nuclear magnetic resonance (^1^H NMR). Spectra were collected using a Brüker Avance DPX-400 NMR spectrometer (Milan, Italy) operating at 400.13 MHz. High Performance Liquid Chromatography (HPLC) analyses were carried out using a Perkin Elmer 250B pump (Waltham, MA, USA) equipped with a Perkin Elmer Flexar UV/Vis LC detector, (Waltham, MA, USA) operating at 425 nm, and a 250 × 4.6 mm^2^ C18 analytical column ODS ultrasphere 5 μm (Beckman Instruments, Brea, CA, USA). The mobile phase was a 60:40 (*v*/*v*) distilled water/acetonitrile-2% acetic acid mixture, filtered through a 0.45 µm nylon membrane and ultrasonically degassed before use; the flow rate was 1 mL min^−1^. Curcumin concentration was determined through a calibration curve with a linear regression coefficient 0.999 in the concentration range 2–20 μg mL^−1^ obtained using an external standard method (Figure S12 in Supplementary Materials).

### 2.3. Synthesis of 6-Deoxy-6-mono-O-(p-toluenesulfonyl)-β-cyclodextrin (β-CD-OTs)

Anhydrous β-cyclodextrin (10.12 g; 8.81 mmol) was suspended in ultrapure water (30 mL) and a 2.5 M aqueous sodium hydroxide (35 mL) was added until complete dissolution of the suspended solid. Toluene-4-sulfonyl chloride (2.59 g; 13.24 mmol) was added in five portions and the formed suspension magnetically stirred for 1.5 h at 21 °C. The suspension was filtered, and the filtrate acidified using a pre-swelled Amberlite IRC-86 resin (11.97 g, 108.33 eq., H^+^-form, exchange capacity 9.05 meq g^−1^). After 35 min, the resin was collected on a 250 µm metal sieve, and the precipitate was recovered from the resulting suspension by centrifuging, then washed with water. The final product was retrieved as a white powder and dried under vacuum until constant weight (3.51 g). Yield: 30%.

### 2.4. Synthesis of 6-Deoxy-6-thioacetic-β-cyclodextrin (β-CD-SAc)

Dry β-CD-OTs (2.51 g, corresponding to 1.95 mmol reactive function) was dissolved in DMF (10 mL) under nitrogen and potassium thioacetate (0.34 g, 2.98 mmol) was added. The reaction mixture was stirred at 20 °C for 3 days under nitrogen atmosphere. The reaction mixture was poured into acetone (200 mL) under vigorous stirring. The whitish precipitate obtained was filtered at reduced pressure and dried under vacuum until constant weight (2.11 g). Yield: 89%.

### 2.5. Synthesis of 6-Deoxy-6-mercapto-β-cyclodextrin (β-CD-SH)

6-Deoxy-6-thioacetic-β-cyclodextrin (2.40 g, corresponding to 2.01 mmol reactive function) and lithium hydroxide monohydrate (0.23 g, 5.55 mmol) were dissolved in de-oxygenated water (22 mL). The reaction mixture was maintained under inert atmosphere and gently magnetically stirred at 50 °C for 24 h. After this time, it was cooled down to room temperature, and the pH adjusted to 2 with 6 M HCl. The reaction mixture was precipitated in de-oxygenated 2-propanol (176 mL), left at 5 °C overnight, and the supernatant partially removed by decanting. The obtained slurry was filtered under reduced pressure while keeping the whole apparatus under nitrogen. The final product, a white powder, was dried under reduced pressure to constant weight (1.51 g). Yield: 65%. Purity (-SH content): 62%.

The -SH content was determined by iodimetric titration using a 0.088 M I_2_ standard solution.

### 2.6. Synthesis of α,ω-Acrylamide End-Functionalized PAA Oligomers

The synthetic procedures adopted were previously described [26]. Briefly:

*MArM*. *N*,*N*′-methylene-bis-acrylamide (26.48 g, 171.0 mmol) and 2,6-di-*tert*-butyl-*p* -cresol (0.53 g, 2.4 mmol) were dissolved in a 6:1 *v*/*v* water/methanol mixture (140 mL), and maintained under stirring at 70 °C. An aqueous L-arginine solution (3.08 g, 17.4 mmol in 25 mL water) was added dropwise and the resultant solution stirred at 70 °C for 1 day. The reaction mixture was maintained for 3 days at the same temperature without stirring and then cooled down to ~0 °C with an ice bath. The solid precipitate was removed by filtering and the supernatant solution freeze-dried. The solid product was dissolved in a 10:1 *v*/*v* methanol/water mixture (25 mL) and the solution poured in acetone (300 mL). MArM was retrieved as a white powder by centrifuging and filtering, and finally dried under vacuum to constant weight (4.06 g). Yield 71%, purity ≥ 98%, as determined by ^1^H NMR.

*ArMAr*. L-arginine (5.43 g, 30.6 mmol) was dissolved in water (60 mL) at room temperature. *N*,*N*′-methylene-bis-acrylamide (2.39 g, 15.3 mmol) and 2,6-di-*tert*-butyl-*p*-cresol (0.06 g, 0.2 mmol) were dissolved in a 1:3 *v*/*v* water/methanol mixture (9 mL) maintained at 50 °C. The warm solution was added dropwise to the L-arginine solution under stirring and the obtained solution was maintained at room temperature for 24 h. After this time, methanol was removed under reduced pressure and the remaining aqueous solution freeze-dried; yield > 98% (7.57 g), purity ≥ 94.5%, as determined by ^1^H NMR.

*MArMArM*. *N*,*N*′-methylene-bis-acrylamide (6.13 g, 39.4 mmol) and 2,6-di-*tert*-butyl-*p*-cresol (0.144 g, 0.6 mmol) were dissolved in a 1:1 *v*/*v* water/methanol mixture (40 mL) at 40 °C under stirring. An aqueous ArMAr solution (1.96 g, 3.9 mmol in 10 mL water) was added dropwise under stirring, and the reaction maintained at 40 °C for 4 days. The final product was retrieved as described for MArM. Yield 63% (1.99 g), purity ≥ 99%, as determined by ^1^H NMR.

### 2.7. Synthesis of P3 and P5

6-Deoxy-6-mercapto-β-cyclodextrin with (0.99 g; corresponding to 0.53 SH meq) and MArM (0.12 g; 0.25 mmol) were added to water (0.740 mL) under stirring in nitrogen atmosphere. The reaction was maintained under stirring at 50 °C for 3 days. The raw product was filtered through a 0.45 μm HPLC filter and then ultrafiltered through an Amicon^®^ Millipore ultrafiltration system using a regenerated cellulose membrane with nominal molecular weight cut-off 3000.

P5 was synthesized as described for P3, by reacting 6-deoxy-6-mercapto-β-cyclodextrin (1.01 g, corresponding to 0.54 SH meq) and MArMArM (0.22 g, 0.27 mmol) in water (0.800 mL).

### 2.8. Preparation of Curcumin Complexes at Different pH Levels and Curcumin/P3 (or P5) Molar Ratios

*2:1 Curcumin/P3 (or P5) molar ratio*

An aqueous solution at pH 7.4 (or a phosphate buffer solution at pH 4.5) of P3 (or P5) (10 mg mL^−1^) was prepared and curcumin added in a 2:1 molar ratio with respect to P3 (or P5), corresponding to 1 mole curcumin/β-CD unit. The mixture was stirred at room temperature for 24 h in the dark and then centrifuged. The curcumin complex was recovered by centrifugation and lyophilization of the supernatant. The lyophilized product was stored at 4 °C.

*3:1 Curcumin/P3 (or P5) molar ratio*

The capability of P3 and P5 to increase the apparent solubility of curcumin was studied in pH 7.4 and pH 4.5 buffer solutions at 25 ± 1 °C. Excess amount of curcumin (15 mg) was introduced in a 10 mL vial containing 5 mL P3 or P5 aqueous solutions with concentration 10 mg mL^−1^. The vials were placed on a shaker for 24 h at 25 ± 1 °C in the dark until saturated solutions were obtained. The samples were then centrifuged at 5000 rpm and the supernatants filtered through a 0.45 μm filter. The filtrates were extracted with chloroform as described in Section 2.9. After chloroform evaporation, the solid samples were dissolved in methanol and the curcumin concentration determined by HPLC analysis.

### 2.9. Determination of Drug Loading

Each curcumin complex (10 mg) was dissolved in water (1 mL) and the solution extracted with chloroform (3 × 1 mL). The collected organic phases were dried by fluxing nitrogen at room temperature. The recovered curcumin was dissolved in methanol and diluted 1:10 in the HPLC mobile phase and analyzed.

### 2.10. Evaluation of Curcumin Photostability in the Complexes

The photostability of the P3- and P5-curcumin complexes after exposure to sunlight was evaluated with respect to curcumin in methanol solution. A stock solution of curcumin was prepared in methanol at the concentration of 0.1 mg mL^−1^ and exposed to light for 72 h. The curcumin complexes were dispersed in water at the concentration of 10 mg mL^−1^ and irradiated to sunlight for the same time. After irradiation, the curcumin solution was diluted in methanol and analyzed by HPLC analysis, while the curcumin was extracted with chloroform from the curcumin complexes, as described in Section 2.9.

### 2.11. In Vitro Release of Curcumin

The in vitro release of curcumin was monitored using the custom-made multi-compartment rotating apparatus shown in Supplementary Materials (Figure S11). This consisted of 8 cylindrical cells, each separated into two compartments by a dialysis membrane with molecular weight cut off 1000. One of the two compartments was filled through a small channel with 1 mL donor phase, consisting of a 20 mg mL^−1^ curcumin complex aqueous solution, using a syringe. The recipient compartment was filled with 1 mL of a pH 6.8 phosphate buffer solution added with 0.1% sodium lauryl sulfate. After filling, the communication channels with the donor and receiving compartments were closed with plastic caps, and the apparatus was kept in slow rotation. The recipient phase was withdrawn and replaced with fresh medium after fixed time intervals. The collected samples were analyzed by HPLC.

The mechanism of curcumin release from P3- and P5-complexes was studied by applying different mathematical models (Equations (1)–(5)):

Zero-order model [31]:

(1) F=kt

First-order model [32]:

(2) ln(1−F)=−kt

Higuchi model [33]:

(3) $$F=kt^{1/2}$$

Hixon–Crowell model [34]:

(4) $$1-{\left(1-F\right)}^{1/3}=kt$$

Korsmeyer–Peppas model [35]:

(5) $$F=kt^{n}$$

where *F* is the drug release fraction and *t* the release time. In all instances, the squared correlation coefficients (*R^2^*) and slope (rate constant, *k*) were obtained by applying a linear regression method.

In the zero-order model (Equation (1)), drug release is proportional to time and totally independent of drug concentration, as typical of delivery systems loaded with scarcely water-soluble drugs.

In the first-order model (Equation (2)), the diffusion rate is proportional to drug concentration. The first-order model is often used to describe the release of hydrophilic drugs dispersed in porous matrices [36].

The Higuchi model (Equation (3)) is based on Fick’s Law, where the release occurs by the drug diffusion within the delivery system.

The Hixon–Crowell model (Equation (4)) can be applied to the delivery systems whose drug release rate is proportional to the surface area of the system, such as the erosion-dependent release systems.

The Korsmeier–Peppas model (Equation (5)), used when the release mechanism deviates from Fickian diffusion, as a result of an anomalous transport, also called non-Fickian release. In this model the release exponent (*n*) characterizes different release mechanisms. If the *n* < 0.5, the release mechanism follows Fickian diffusion, if 0.5 < *n* < 1 the release mechanism is defined non-Fickian.

### 2.12. Molecular Mechanics (MM) and Molecular Dynamics (MD) Simulations

MM and MD methods were applied to the study at the atomistic level of P3- and P5-curcumin complexes or aggregates obtained at pH 4.5 and pH 7.4 by making twenty curcumin molecules interact with ten P3 or P5 molecules, that is, in a 1:1 curcumin/β-cyclodextrin stoichiometric ratio. These simulations are performed using the consistent valence force field (CVFF) [37] and the Materials Studio packages [38]. The simulation protocol is the same adopted in the previous work [20,21,22,23,24,25]. This protocol provides (i) the initial energy minimization, (ii) MD runs until the equilibrium state was achieved (iii) final geometry optimizations of the final configuration and of some different configurations assumed by the system during MD run. The curcumin, P3 and P5 structures were generated using the Module Builder [38]. The conformational study of P3 and P5 at the two pH levels of interest was initially performed in order to find the most stable geometries. In order to study the possible aggregation process of P3 or P5 carrier molecules and curcumin molecules, four cubic cells with 220 Å side were generated (Figure S14), including twenty curcumin molecules and ten P3 or P5 molecules with the appropriate charge according to the considered pH. The curcumin, P3 and P5 molecules were initially placed in the simulation box in a random distribution in order to study the interactions without assuming any a priori inclusion complexes or just formed nanoaggregate. It is important to underline that this random arrangement in all simulation cells implies starting from a situation in which all the molecules are well solubilized in solution. The simulations were performed in implicit water using a distance-dependent dielectric constant with periodic boundary conditions. All energy minimizations were carried out up to an energy gradient < 4 × 10^−3^ kJ mol^−1^ Å^−1^. The MD run were performed in an NVT canonic ensemble controlled using a Berendsen thermostat at a constant temperature (300 K). The integration of dynamical equations was carried out with the Verlet algorithm using a time step of 1 fs and the instantaneous coordinates were periodically saved for further analysis. The MD runs lasted for 50 ns, and every 50 ps the conformations were saved and analyzed. During the MD runs, the time evolution of the potential energy, the van der Waals and the electrostatic components together with the variation of the Radius of gyration of all the molecules in the simulation cell were calculated. The theoretical discussion will be presented in Section 3.5.

## 3. Results and Discussion

### 3.1. Synthetic Procedures

Two polyamidoamine (PAA) oligomers bearing β-cyclodextrin units at both ends, coded P3 and P5 (Figure 1), with similar structure but different distance and cooperation potential between the two terminal β-CD units, were synthesized as water-soluble pH-sensitive curcumin complexing agents.

P3 and P5 are amphoteric and exhibit a pH-dependent charge distribution, as exemplified in Table 1. In particular, they feature fully protonated arginine groups at pH < 10 and fully dissociated carboxyl groups at pH > 4. The protonation degree of the *tert*-amine group present in the main chain in the physiological relevant pH range 4.5–7.4 is pH-dependent. The presence, in the structure of P3 and P5, of two β-CD units containing hydrophobic cavities connected by an ionic oligomeric chain suggests a high potential for establishing stable interactions with curcumin, a hydrophobic molecule containing functions capable of forming polar hydrogen bonds.

**Scheme 1:**
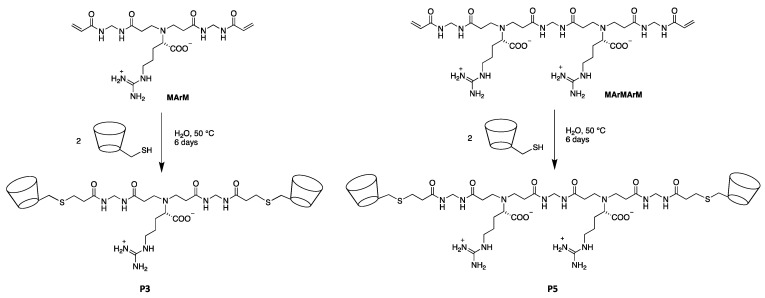
Synthesis of the P3 and P5 β-cyclodextrin α,ω-end-functionalized PAA oligomers.

P3 and P5 were obtained by the thio-Michael addition of 6-deoxy-6-mercapto-β-cyclodextrin (β-CD-SH) with α,ω-end-acrylamide-functionalized PAA oligomers of different polymerization degree, that is, a trimer, MArM, and a pentamer, MArMArM (Scheme 1).

**Scheme 2:**
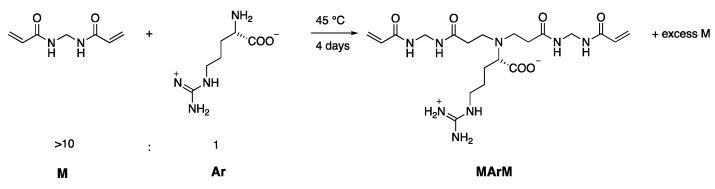
Synthesis of the α,ω-acrylamide-end-functionalized MArM trimer.

**Scheme 3:**
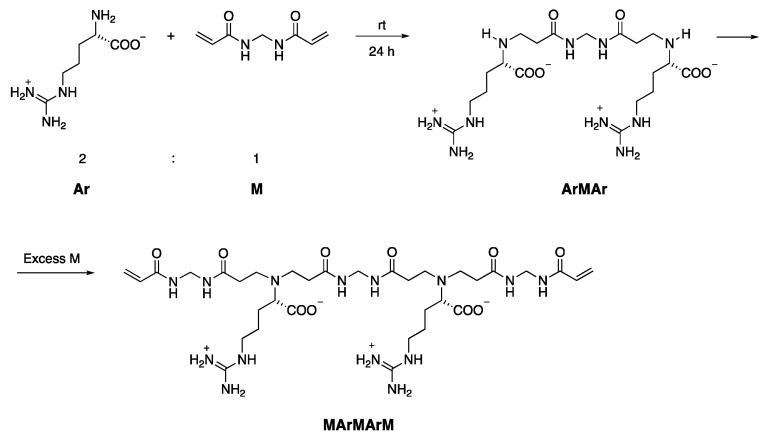
Synthesis of the α,ω-acrylamide-end-functionalized MArMArM pentamer.

MArM and MArMArM were, in turn, obtained as previously described through the controlled polyaddition reaction of *N*,*N*′-methylene-bis-acrylamide with L-arginine (Scheme 2 and Scheme 3) [30].

**Scheme 4:**
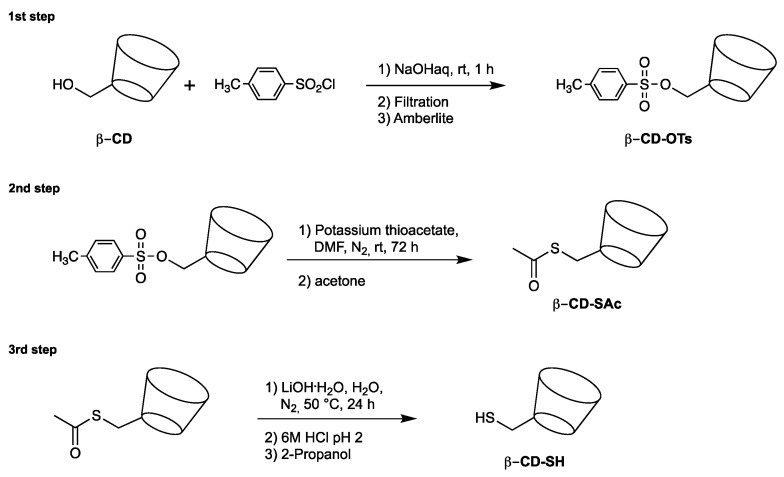
Synthesis of 6-deoxy-6-mercapto-β-cyclodextrin (β-CD-SH).

β-CD-SH was prepared as previously described by a three-step synthetic pathway (Scheme 4) [40]. In the first step, 6-deoxy-6-tosyl-β-cyclodextrin (β-CD-OTs) was synthesized by reacting β-cyclodextrin with tosyl chloride [41]; β-CD-OTs was then reacted with potassium thioacetate to give β-CD-SAc; β-CD-SH was finally obtained by the alkaline hydrolysis of β-CD-SAc with lithium hydroxide at 50 °C. The β-CD-SH content was determined iodimetrically using a standard KI_3_ solution. Both P3 and P5 were remarkably soluble in water, exhibiting a solubility higher than 200 mg mL^−1^ at 20 °C, that is, more than two orders of magnitude higher than that of β-CD. Both P3 and P5, as well as their intermediates, were characterized by ^1^H NMR and FT-IR spectroscopies. The ^1^H NMR spectra of P3 and P5, run in D_2_O using a 400 MHz apparatus, as well as those of their precursors are shown in Figures S1–S8 with the related assignments. The FT-IR spectra of P3 and P5 oligomers are shown in Figures S9 and S10. All spectral data fully agreed with the proposed structures.

### 3.2. P3- and P5-Curcumin Complexes

P3- and P5-curcumin complexes were prepared by the freeze-drying method [42,43] at two physiologically relevant pH levels, namely the plasma pH 7.4 and the typical pH inside lysosomes 4.5. In all four cases, the established procedure consisted of incubating an aqueous solution of P3 (or P5) with curcumin in a 2:1 or 3:1 molar ratio with respect to P3 (or P5), corresponding to 1 mole curcumin and 1.5 mole curcumin/β-CD unit, respectively, and then stirring the obtained mixture for a prolonged time. After separating the solid, the supernatant was freeze-dried yielding the curcumin complex as a water-soluble yellow powder. The curcumin loadings of all complexes were determined by extracting the dry powders with chloroform to quantitatively remove curcumin and retrieved the curcumin by solvent evaporation. The amount of extracted solid curcumin was quantitatively determined by both HPLC analysis and by UV spectroscopy carried out in chloroform solution at 416 nm. The determined loading values, reported in Table 2, show that curcumin loadings of P3 and P5 ranged approximately between 0.7 and 1.5% and that P3 loading was invariably lower than that of P5. The FT-IR/ATR spectra of curcumin-loaded P3 and curcumin-loaded P5, as well as those of free curcumin and free P3 and P5, are shown in Figures S9 and S10. Due to the relative low curcumin loading, no significant differences were observed between the spectra of P3 and P5 and those of their curcumin complexes.

Both P3- and P5-curcumin freeze-dried complexes were easily re-dissolved in water forming stable solutions with concentrations up to approximately 3.6 mg mL^−1^, with shelf-stability up to at least 30 days, as revealed by the absence of precipitation phenomena within this time period. The apparent solubility of curcumin ranged approximately from 20 to 55 μg mL^−1^, with an increase in the apparent drug solubility between approximately 1800 to 4600 times compared with that of free curcumin, which, according to the literature, is in the range 0.011 μg mL^−1^ [13].

### 3.3. Enhanced Curcumin Photostability by Complexation with P3 and P5

Free curcumin as methanol solution underwent complete photodegradation within 72 h irradiation to daylight. Photodegradation was rapid and reached more than 50% within 4 h. The P3- and P5-curcumin complexes were remarkably more stable both at pH 7.4 and pH 4.5. In fact, only a 15% degradation was observed in water after 72 h under the same irradiation conditions (Table 3).

### 3.4. Curcumin Release Studies

**Figure 2:**
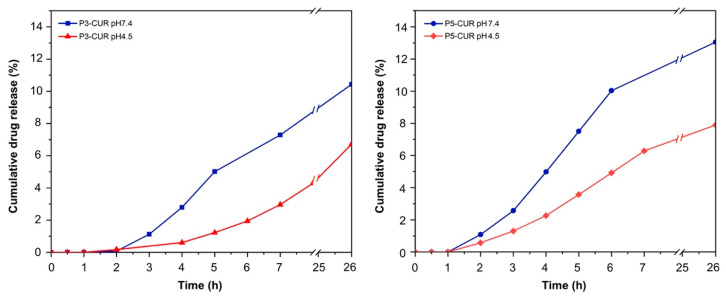
In vitro release curves of curcumin from the P3- and P5 complexes.

The in vitro release profiles of curcumin from the P3- and P5 complexes were determined at pH 7.4, the plasma pH, and at pH 4.5, the pH of the intracellular environment. In all instances, the release curves (Figure 2) showed an induction period of approximately 2 h for the P3-curcumin complexes and of approximately 1 h for the P5 ones. The absence of any burst effect and the delay observed in curcumin release suggested that the drug molecules are stably encapsulated in the core of the P3- and P5 complexes, and not simply adsorbed on the outer surface, and let preliminarily envisage a diffusion-controlled release mechanism.

The kinetic data were analyzed using the five mathematical models described by Equations (1)–(5), Section 2.11. The experimental curves were fitted using all five models from 0 to approximately 7 h. The fitting curves are reported in Figure S13, whereas the values of the kinetic constants and the corresponding *R*^2^ values are shown in Table 4. The experimental data were nicely fitted by Equations (1) and (2), corresponding to zero-order and first-order release kinetics, and the related fitting curves gave maximal *R^2^* values, confirming the occurrence of a diffusive release mechanism. Curcumin release was clearly pH-dependent, as indicated by the significantly higher release rate constants observed at pH 7.4 compared to pH 4.5 (Table 4). Moreover, at the same pH, the release rate from the P5-complex was higher than that from the P3 one. MM and MD studies (see below Section 3.5) allowed to correlate the different release rates observed to the different conformational states of the P3- and P5-curcumin complexes at the two pH levels considered.

### 3.5. MD Studies of P3- and P5-Curcumin Aggregation Process in a 1:2 Stoichiometry

**Figure 3:**
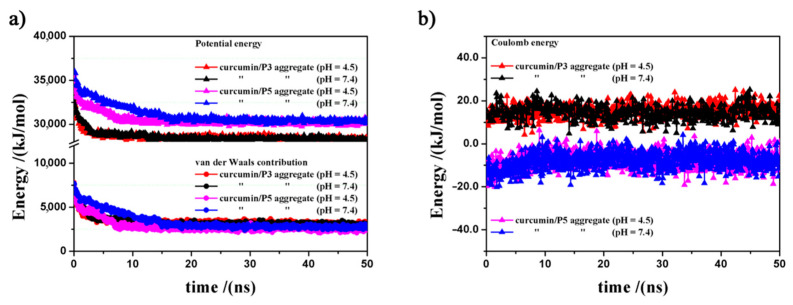
Potential energy, van der Waals (**panel a**) and electrostatic contributions (**panel b**) calculated during the MD runs related to the curcumin/P3 and curcumin/P5 aggregates at pH 4.5 and pH 7.4.

Using a simulation protocol proposed in previous work [20,21,22,23,24,25], after the initial energy minimizations of the simulation cells having 20 curcumin molecules and 10 P3 or P5 molecules in the initial random arrangements, four different MD runs of the systems at two different pH values (pH 7.4 and 4.5) lasting 50 ns were performed. It is worth underlining here that the random arrangement in the simulation cell corresponds to the hypothesis of initial complete solubilization in water of all involved molecules. The potential energy, the van der Waals and electrostatic components calculated during the MD runs are shown in Figure 3. Both P3 and P5 quickly form aggregates with the curcumin molecules. In particular, the kinetics of the drug/carrier aggregation process are faster when the P3 molecules are involved, particularly so at pH 4.5. The driving forces for aggregation are the van der Waals interactions (Figure 3a), which remain stable throughout the entire MD run. Furthermore, during MD runs the P3 carrier displays electrostatic interactions that fluctuate around an average value, while for P5 they initially increase due to some repulsion when the molecules form the first complexes (Figure 3b) but the H-bonds among the carrier molecules stabilize the aggregates.

In line with the observed experimental data, the highest stability of the formed nanoaggregates for the two different carriers was calculated at pH 4.5. The stabilization with respect to 20 drug molecules and 10 carrier molecules considered in the most stable geometry in the isolated state both for P3 and P5 carrier is larger by 5859 and 6214 kJ mol^−1^ at acid pH 4.5, and by 5810 and 6003 kJ mol^−1^ at pH 7.4, respectively (Table 5).

The stability of the optimized geometries assumed at the end of the MD run when the equilibrium state is achieved is higher at pH 4.5 than at pH 7.4 for both the P3- and P5-curcumin aggregates. In particular, the P3-based aggregate is 84.36 kJ/mol more stable at pH 4.5 than at pH 7.4, whereas the P5-based aggregate is 204.53 kJ mol^−1^ more stable at pH 4.5 than at pH 7.4. H-bonds between β-CDs stabilize the aggregates.

**Figure 4:**
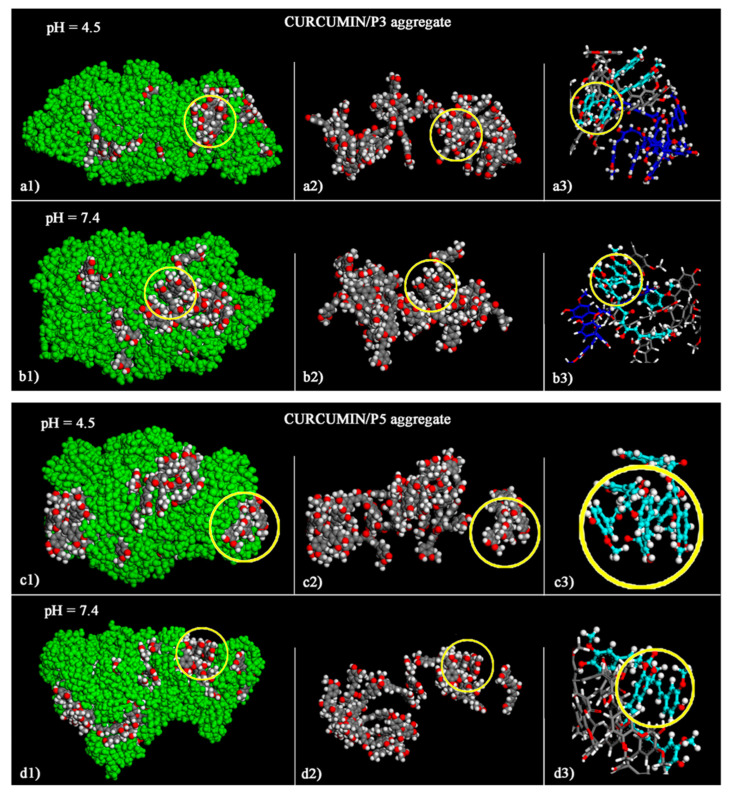
Optimized geometries of the curcumin/P3 aggregates at the end of MD run at pH 4.5 (**a1**–**a3**) and at pH 7.4 (**b1**–**b3**), and of the curcumin/P5 aggregates at pH 4.5 (**c1**–**c3**) and at pH 7.4 (**d1**–**d3**). On the left-hand panels (**a1**,**b1**,**c1**,**d1**) the atoms of P3 and P5 are all colored in green. In the same panels, the color codes of the curcumin molecules are as follows: carbon, grey; oxygen, red; hydrogen, white. For clarity, the carbon atoms of P3 and P5 are not shown in the mid panels (**a2**,**b2**,**c2**,**d2**). In order to provide a clearer picture of the π–π interaction among curcumin molecules, the sticks and balls structures of all aggregates are shown on the right-hand panels (**a3**,**b3**,**c3**,**d3**).

During the MD runs, all aggregates showed that the curcumin molecules are not completely located inside the aggregates, but are partially exposed on the outer surface, as shown in the optimized geometries calculated at the end of the MD runs shown in Figure 4. It may be observed that the curcumin molecules interact with each other thanks to the hydrophobic *π–π* interactions (Figure 4(a3)–(d3)) but in all cases they are partly dispersed in the nanoaggregates thanks to favorable van der Waals interactions with the carrier molecules. For both P3 and P5, this dispersion in the formed aggregate is influenced by the solution pH. For instance, at pH 4.5, only one of the twenty curcumin molecules in the simulation cell does not expose atoms on the aggregate external surface, whereas at pH 7.4, only two curcumin molecules do not expose atoms on the aggregate external surface. Interestingly, at both pH values curcumin forms five 1:1 host–guest inclusion complexes with the β-CD cavities of P3, whereas in the case of P5 two and three curcumin molecules form 1:1 inclusion complexes with the hydrophobic β-CD cavities, at pH 4.5 and pH 7.4, respectively.

In the aggregates, the curcumin molecules involved in host–guest inclusion complexes, compared to those exposed on the outer surface, probably influence the drug release kinetics by slowing it down. Indeed, it was previously observed in the case of piroxicam/β-CD nanosponges [23] that drug molecules located on the outer surface were released faster and those forming host–host inclusion complexes more slowly.

Finally, it is worth observing here that all curcumin/P3 and curcumin/P5 aggregates did not show any degradation process during the entire MD run and were stable with the drug molecules dispersed in the nanoaggregates (Figure S14). This aspect confirms, in particular, the high stability of these systems, as found experimentally.

**Figure 5:**
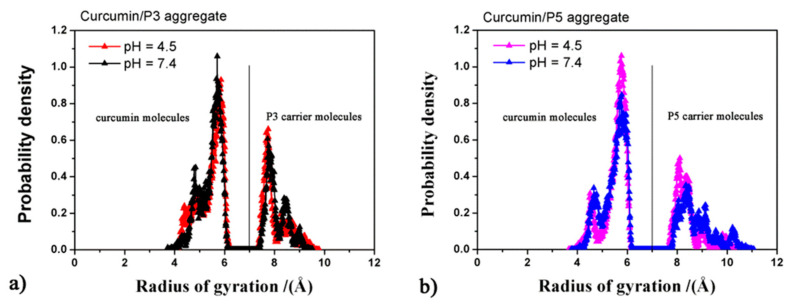
Radii of gyration of all molecules calculated during MD runs for the curcumin/P3 aggregates (**panel a**) and of the curcumin/P5 aggregates (**panel b**).

The conformation of all curcumin and P3 or P5 molecules in the aggregates were further analyzed by calculating the distribution of their radii of gyration (*R_g_*) during the MD runs for all saved frames (Figure 5). As regards curcumin, elongated conformations (*R_g_* ≅ 5.70 Å) were frequently observed, whereas more compact conformations (*R_g_* ≅ 4.5 Å), likely due to the folding inducing *π–π* interactions of the aromatic rings, were less frequently detected. The radius of gyration of the P3 molecules range from 7.3 to 9.7 Å, whereas those of P5 exhibit slightly larger values, from 7.5 to 11 Å. The solvent accessible surface area of the P3- or P5-curcumin aggregates featured a similar and less extended surface area at pH 4.5 (Table 5) with respect to those observed at pH 7.4. The higher accessibility of the curcumin aggregates at physiological pH can explain the faster release observed at this pH (Table 5).

## 4. Conclusions

The aim of the research reported in this paper was to design oligomeric, water-soluble and biocompatible carriers for delivering curcumin, a well-known anticancer drug, whose employment is severely hampered by its extremely low water solubility (0.011 μg mL^−1^). In particular, two amphoteric polyamidoamine oligomers bearing at both ends β-cyclodextrin units, namely P3 and P5, were proposed as pH-sensitive carriers. The polyamidoamine oligomeric precursors, a trimer (P3) and a pentamer (P5), were obtained from a controlled synthesis involving L-arginine and *N*,*N*′-methylene-bis-acrylamide. P3 and P5 proved able to encapsulate curcumin at the two physiologically relevant pH levels of 7.4 and 4.5. After lyophilization, their curcumin complexes gave stable water solutions. The apparent solubility of P3- and P5-encapsulated curcumin ranged between 20 and 51 μg mL^−1^, that is, it was three orders of magnitude higher than the water solubility of the free drug. The analysis of the drug release profiles at pH levels 4.5 and 7.4 suggested a diffusive release mechanism and indicated a faster release rate at pH 7.4 than at pH 4.5. Both P3- and P5-encapsulated curcumin were significantly more photostable than free curcumin.

A theoretical study based on MM and MD simulations allowed to better understand at the atomistic level important aspects related to the conformation of the curcumin, P3 and P5 molecules in the formed drug/carrier nanoaggregates. Indeed, they allowed us to explain the observed results in terms of encapsulation efficiency, surface interaction and aggregate stability and to compare the drug release rates observed in the selected experimental conditions. The driving forces for aggregation were the favorable drug/carrier van der Waals interactions. H-bonds between β-CDs and *π–π* interactions among curcumin aromatic rings stabilized the formed nanoaggregates that were stable throughout the entire MD run, with no degradation. Interestingly, both carriers were more stable at pH 4.5 than at pH 7.4 and the aggregation kinetics were faster at pH 4.5. In all cases, the curcumin molecules were partially exposed on the outer surface and partially incorporated into the aggregate core. In particular, the P3-curcumin aggregates displayed five curcumin molecules forming host–guest inclusion complexes with the hydrophobic β-CD units, whereas in the P5-curcumin aggregates fewer drug molecules were encapsulated at both pH values. The different van der Waals interactions, the different solvent accessible surface areas of the P3- and P5-aggregates and the higher accessibility of the curcumin aggregates at pH 7.4 can explain the faster release observed at this pH.

## Figures and Tables

**Table 1:** pH-dependent ionic species distribution in the repeat units of P3 and P5 ^a^.

pH	COO^−^ Units (%)	Guanidinium Pendant Units (%)	Protonated *Tert*-Amine Units in the Main Chain (%)	Neutral *Tert*-Amine Units in the Main Chain (%)
7.4	100	100	18	82
7.0	100	100	35	65
6.4	100	100	50	50
4.5	100	100	100	0

^a^ Obtained from the following *pK_a_* values of the ionizable functions [39]: *pK_a_*_1_ (COOH): 2.31; *pK_a_*_2_ (*tert*-amine group in the main chain): 6.43; *pK_a_*_3_ (guanidine): >10.

**Table 2:** Apparent solubility of curcumin in the form of P3- or P5-complex versus solubility of free curcumin.

Sample	pH	Curcumin Loading in the Complex (Weight %)	Curcumin/P3 (or P5) Molar Ratio	Apparent Curcumin Solubility (μg mL^−1^)	Solubility Enhancement ^a^
Curcumin				0.011 ± 0.01	-
P3	4.5	0.70 ± 0.04	2:1	25.2 ± 0.02	2290
P3	7.4	0.90 ± 0.03	2:1	39.2 ± 0.03	3563
P5	4.5	1.36 ± 0.02	2:1	49.0 ± 0.03	4454
P5	7.4	1.52 ± 0.02	2:1	54.7 ± 0.03	4972
P3	4.5	0.57 ± 0.02	3:1	20.8 ± 0.24	1890
P3	7.4	0.58 ± 0.02	3:1	20.5 ± 0.10	1863
P5	4.5	1.13 ± 0.03	3:1	40.7 ± 0.85	4477
P5	7.4	1.41 ± 0.03	3:1	50.7 ± 0.39	4609

^a^ Ratio of the apparent solubilization in water of curcumin in the form of P3 or P5 complex and the water solubility of free curcumin.

**Table 3:** Photodegradation of curcumin versus its P3- and P5-complexes.

Time (h)	Percent Degradation (%)				
	Free Curcumin ^a^	P3 Obtained at pH 4.5 ^b^	P3 Obtained at pH 7.4 ^b^	P5 Obtained at pH 4.5 ^b^	P5 Obtained at pH 7.4 ^b^
2	34.17	-	-	-	-
4	54.66	-	-	-	-
72	100	14.46	15.12	14.35	15.15

^a^ As a 0.1 mg mL^−1^ methanol solution; ^b^ as a 10 mg mL^−1^ aqueous solution.

**Table 4:** Kinetic release constants of curcumin obtained using different mathematical models ^a^.

		F = kt		ln(1 − F) = −kt		F = kt^1/2^		1 − (1 − F)^1/3^ = kt		F = kt^0.6^	
Carrier	pH	k ^b^ (h^−1^)	R^2^	k ^b^ (h^−1^)	R^2^	k ^b^ (h^−1/2^)	R^2^	k ^b^ (h^−1^)	R^2^	k ^b^ (h^−0.6^)	R^2^
P3	7.4	0.015	0.986	0.016	0.986	0.061	0.979	0.063	0.979	0.045	0.979
P3	4.5	0.007	0.978	0.007	0.976	0.028	0.836	0.036	0.836	0.020	0.836
P5	7.4	0.023	0.992	0.024	0.990	0.087	0.972	0.008	0.972	0.064	0.972
P5	4.5	0.012	0.988	0.012	0.986	0.047	0.961	0.004	0.961	0.034	0.961

^a^ Where *F* = fraction of released drug, *t* = time; *k* = kinetic constant; *R*^2^ = squared regression coefficient. ^b^ Error = ± 6%.

**Table 5:** Relative stability with respect to isolated state of 10 curcumin molecules and 20 P3- or P5- carriers, and the solvent accessible surface area (Å^2^) of P3- and P5-curcumin aggregates at different pH values in the optimized geometries at the end of MD runs.

Aggregate	pH	Relative Stability Compared to Isolated State (kJ mol^−1^)	SASA ^a^ (Å^2^)
Curcumin/P3	4.5	−5859	9129
Curcumin/P3	7.4	−5810	9566
Curcumin/P5	4.5	−6214	9231
Curcumin/P5	7.4	−6003	10,059

^a^ Solvent accessible surface area.

## Data Availability

Not applicable.

## Supplementary Materials

The following are available online at https://www.mdpi.com/article/10.3390/polym14153193/s1, Figures S1–S3: ^1^H-NMR spectra of β-cyclodextrin derivatives. Figures S4–S8: ^1^H-NMR spectra of MArM, ArMAr, MArMArM, P3 and P5. Figures S9 and S10: FT-IR/ATR spectra of curcumin-complexes. Figure S11: Scheme of the apparatus used in the in vitro curcumin release studies. Figure S12: Calibration curves. Figure S13: Linear regression by mathematical models of drug release curves. Figure S14: Initial non optimized geometries before the first energy minimization.
