# Supplementary material for: L-Arginine-Derived Polyamidoamine Oligomers Bearing at Both Ends β-Cyclodextrin Units as pH-Sensitive Curcumin Carriers
Source: Polymers (Basel). 2022 Aug 5;14(15):3193. doi: 10.3390/polym14153193 (PMC9371169; doi:10.3390/polym14153193)
Supplement: Supplementary file 1 [file polymers-14-03193-s001.zip › polymers-1857859-supplementary.pdf]

Supplementary Materials

L-Arginine-Derived Polyamidoamine Oligomers Bearing at Both Ends β-Cyclodextrin Units as pH-Sensitive Curcumin Carriers

Sofia Treccani,[1] Jenny Alongi,[1] Amedea Manfredi,[1] Paolo Ferruti,[1] Roberta Cavalli,[2] Giuseppina Raffaini,[3]* Elisabetta Ranucci[1]*

[1] Dipartimento di Chimica, Università degli Studi di Milano, via C. Golgi 19, 20133 Milano, Italy; sofia.treccani@unimi.it (S.T.); jenny.alongi@unimi.it (J.A.); amedea.manfredi@unimi.it (A.M.); paolo.ferruti@unimi.it (P.F); elisabetta.ranucci@unimi.it (E.R.)

[2] Dipartimento di Scienza e Tecnologia del Farmaco, Università degli Studi di Torino, via P. Giuria 9, 10125 Torino, Italy; roberta.cavalli@unito.it (R.C.)

[3] Dipartimento di Chimica, Materiali, e Ingegneria Chimica "Giulio Natta", Politecnico di Milano, Piazza L. Da Vinci 32, 20131 Milano, Italy; giuseppina.raffaini@polimi.it (G.R.)

* Correspondence: elisabetta.ranucci@unimi.it (E.R.); Tel.: +39–02-50314132; giuseppina.raffaini@polimi.it (G.R.); Tel.: +39-02- 23993068.

Figures S1-S3: ^{1}H-NMR spectra of β-cyclodextrin derivatives.

Figures S4-S8: ^{1}H-NMR spectra of MArM, ArMAr, MArMArM, P3 and P5.

Figures S9 and **S10:** FT-IR/ATR spectra of curcumin-complexes.

Figure S11: Scheme of the apparatus used in the *in vitro* curcumin release studies.

Figure S12: Calibration curves.

Figure S13: Linear regression by mathematical models of drug release curves.

Figure S14: Initial non optimized geometries before the first energy minimization.

^{1}H-NMR characterization

All β-cyclodextrin derivatives were characterized by ^{1}H-NMR spectroscopy, using a Brüker Avance DPX-400 NMR spectrometer (Milano, Italy) operating at 400.13 MHz. Number of scans 32, relaxation delay, *d1*, 10.0 s, receiver gain automatically measured and set by the instrument. Analyses were conducted in DMSO-d_6.

β-CD-OTs

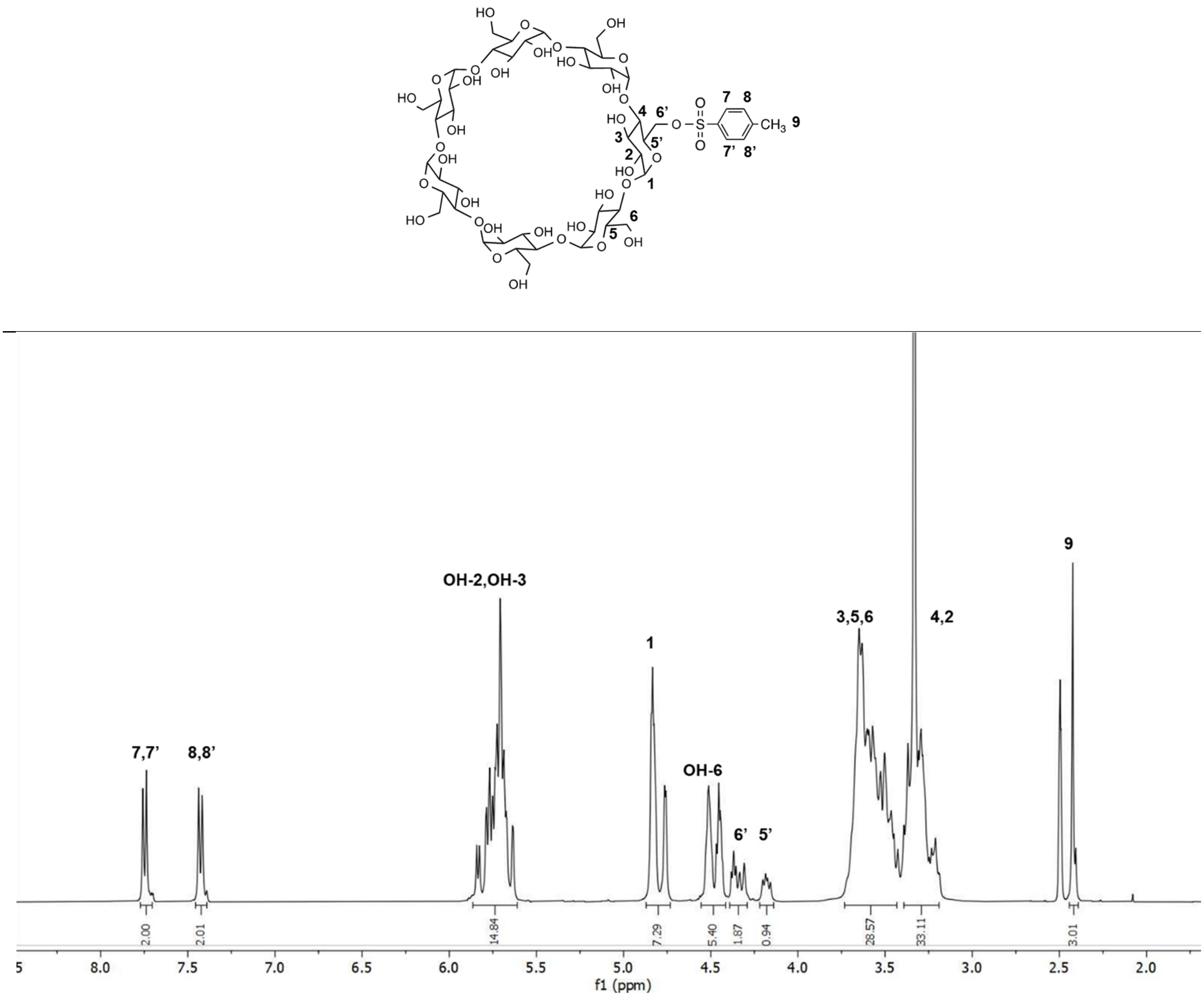

Figure S1. ^{1}H-NMR of β-CD-OTs.

β-CD-SAc

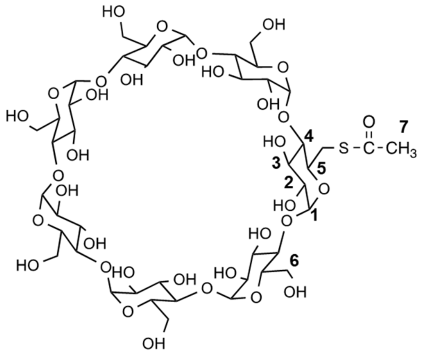

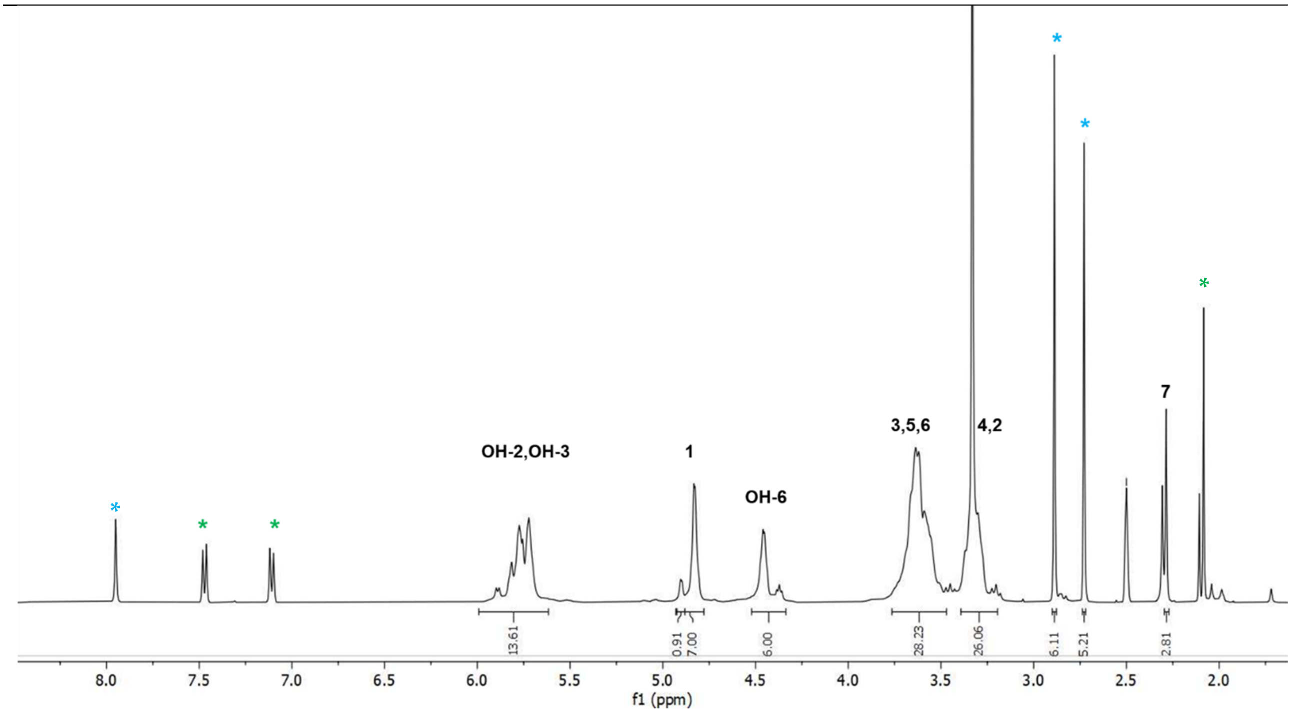

Figure S2. ^{1}H-NMR of β-CD-SAc (*DMF, *Potassium tosylate).

β-CD-SH

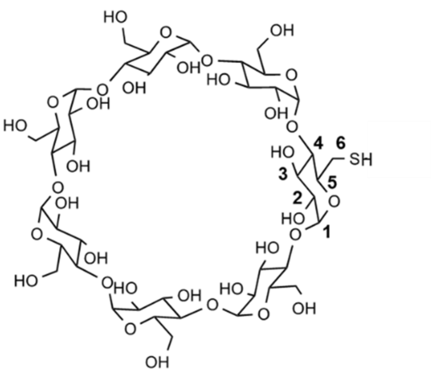

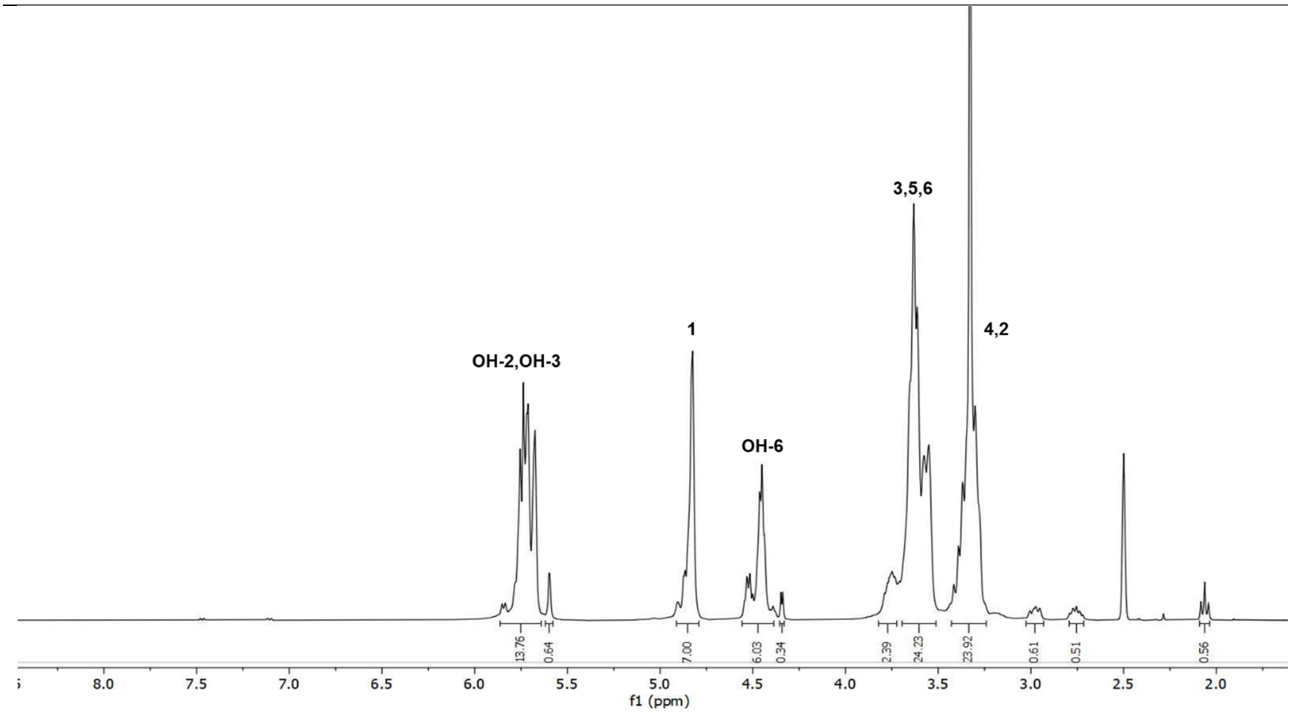

Figure S3. ^{1}H-NMR of β-CD-SH.

All MArM, ArMAr, MArMArM and P3/P5 were characterized by ^{1}H-NMR spectroscopy, using a Brüker Avance DPX-400 NMR spectrometer (Milano, Italy) operating at 400.13 MHz. Number of scans 32, relaxation delay, *d1*, 10.0 s, receiver gain automatically measured and set by the instrument. Analyses were conducted in D_2O.

MArM

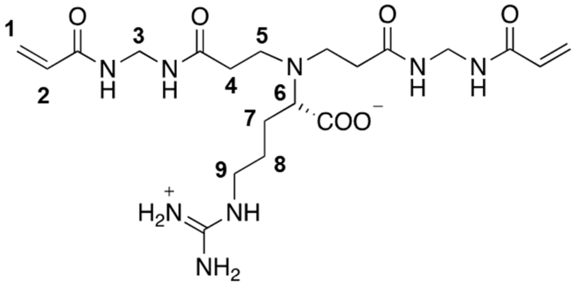

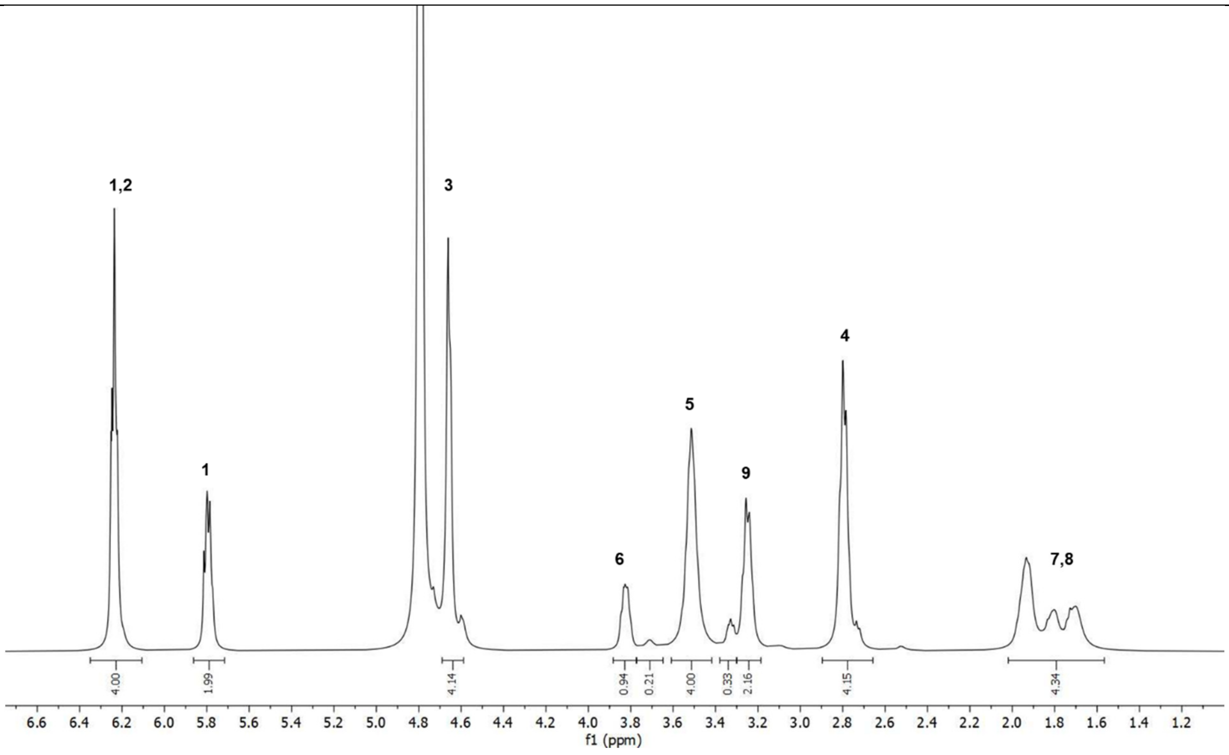

Figure S4. ^{1}H-NMR of MArM in D_2O/DCl at pH 4.5.

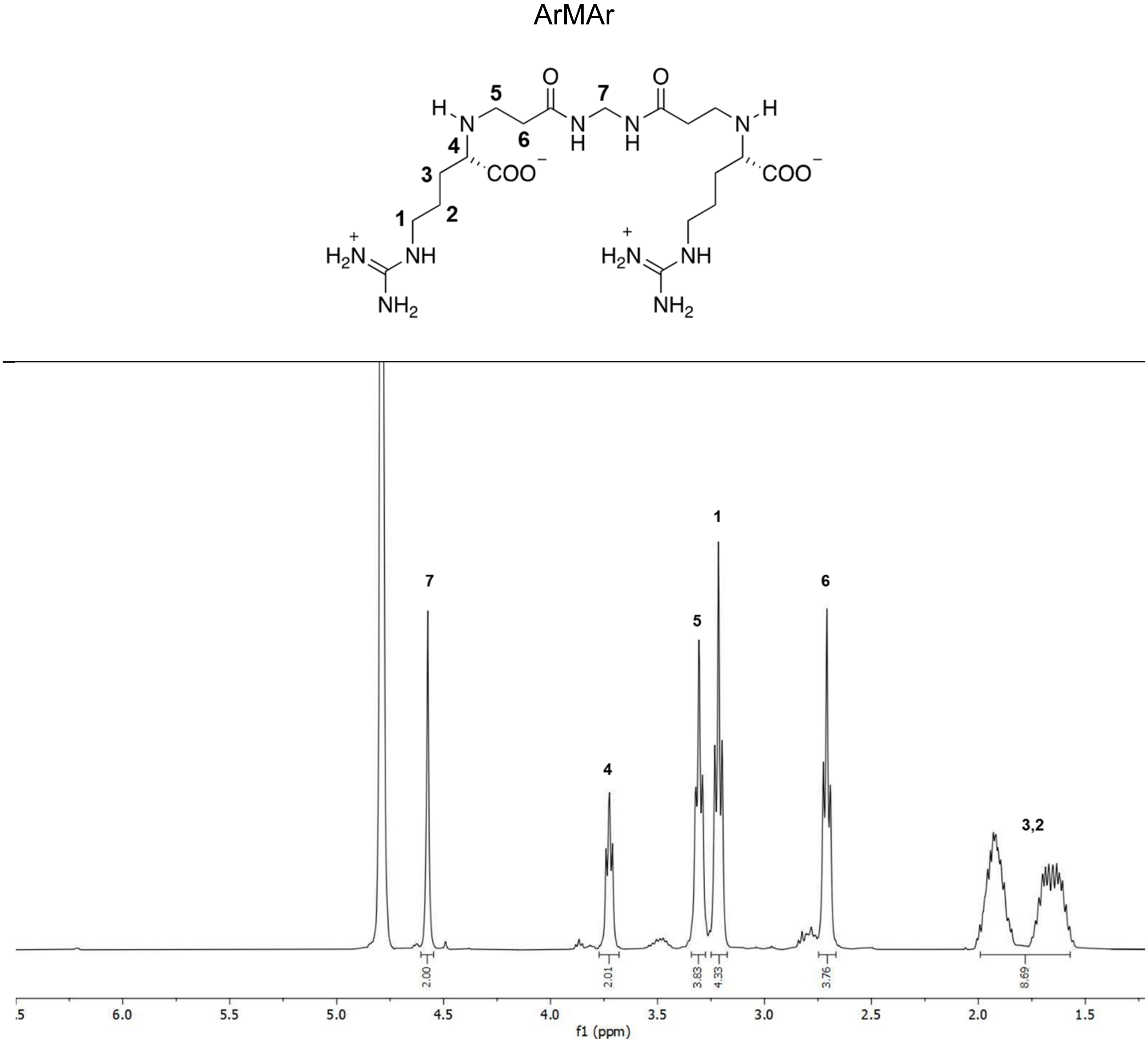

Figure S5. ^{1}H-NMR of ArMAr in D_2O/DCl at pH 4.5.

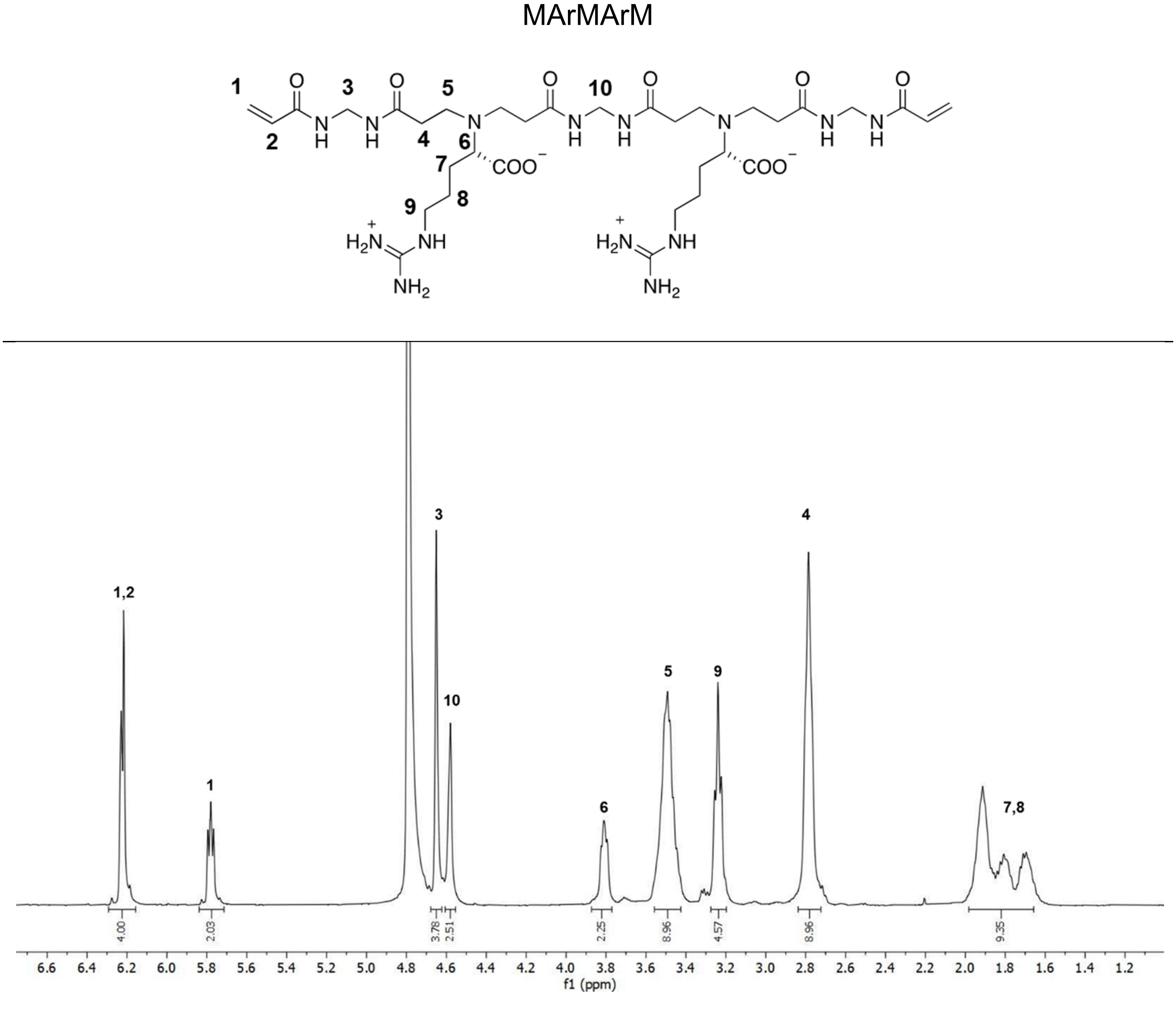

Figure S6. ^{1}H-NMR of MArMArM in D_2O/DCl at pH 4.5.

P3

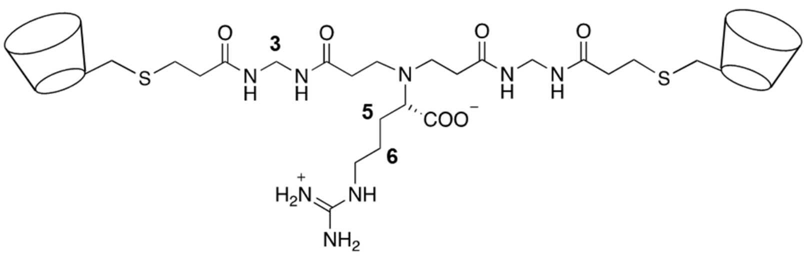

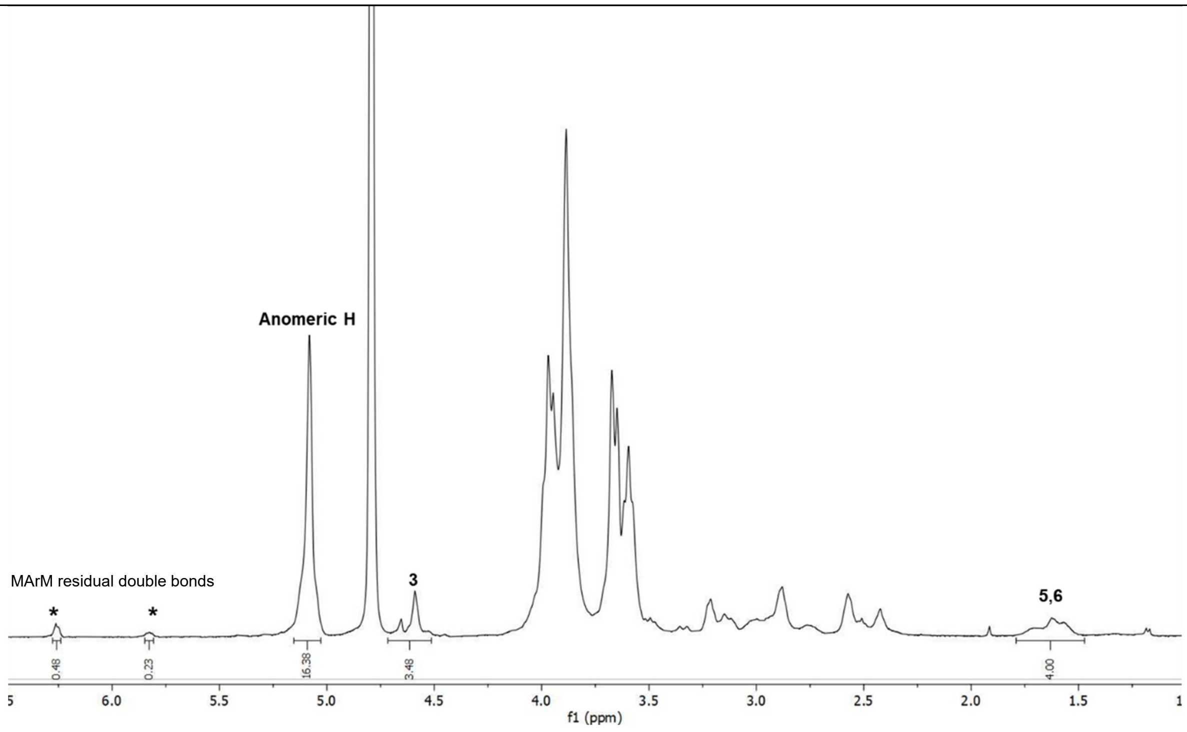

Figure S7 ^{1}H-NMR of P3 in D_2O.

P5

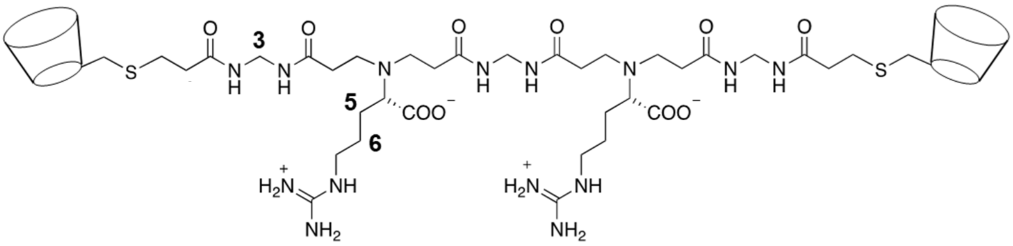

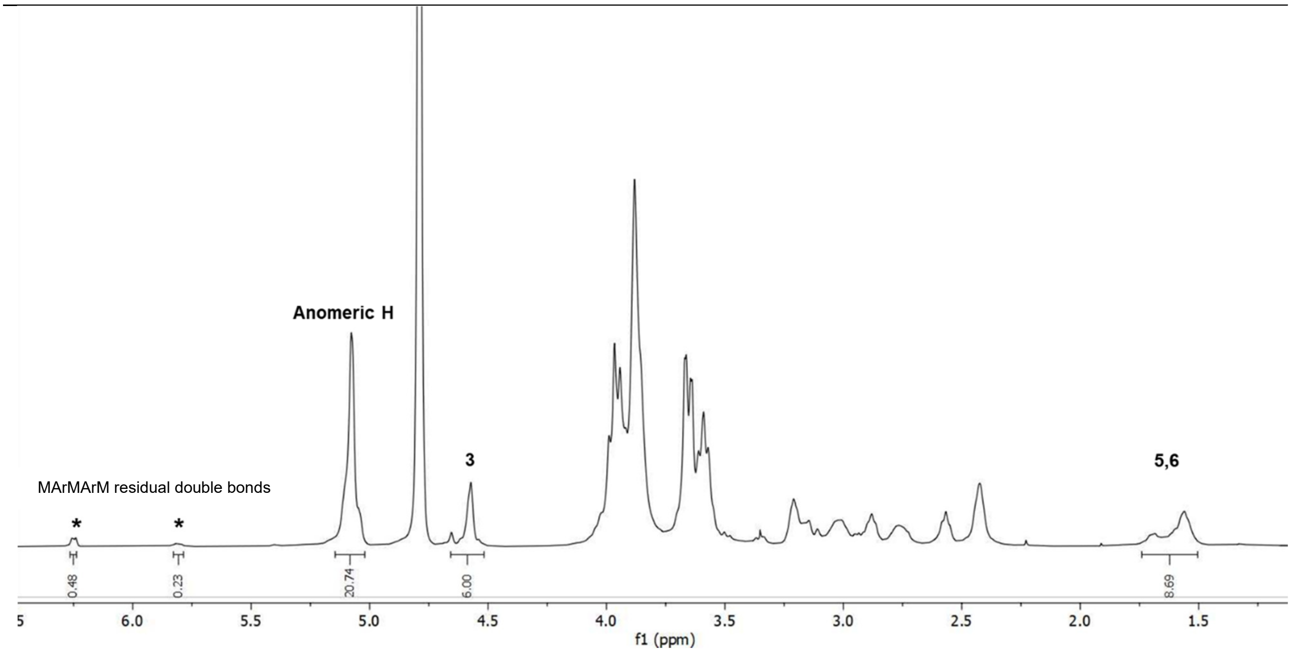

Figure S8. ^{1}H-NMR of P5 in D_2O.

FT-IR/ATR characterization

All curcumin-complexes, P3/P5 and free curcumin were analyzed by attenuated total reflectance (ATR) Fourier transform infrared spectroscopy (FT-IR). FT-IR/ATR spectra were recorded at room temperature, in the 4000 - 500 cm^{-1} wavenumber range, with 32 scans and 4 cm^{-1} resolution using a Perkin-Elmer Frontier FT-IR/FIR spectrophotometer (Milano, Italy), equipped with a diamond crystal characterized by a penetration depth of 1.66 μm.

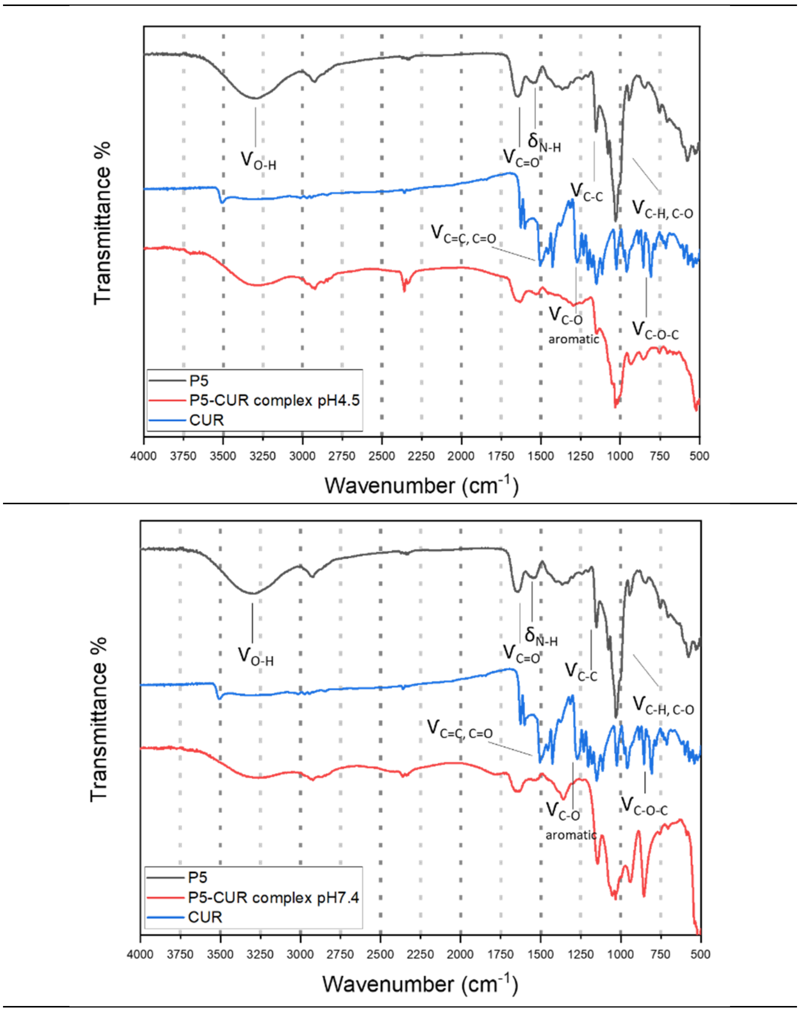

Figure S9. FT-IR/ATR spectra of P5-curcumin complexes at pH 4.5 and 7.4

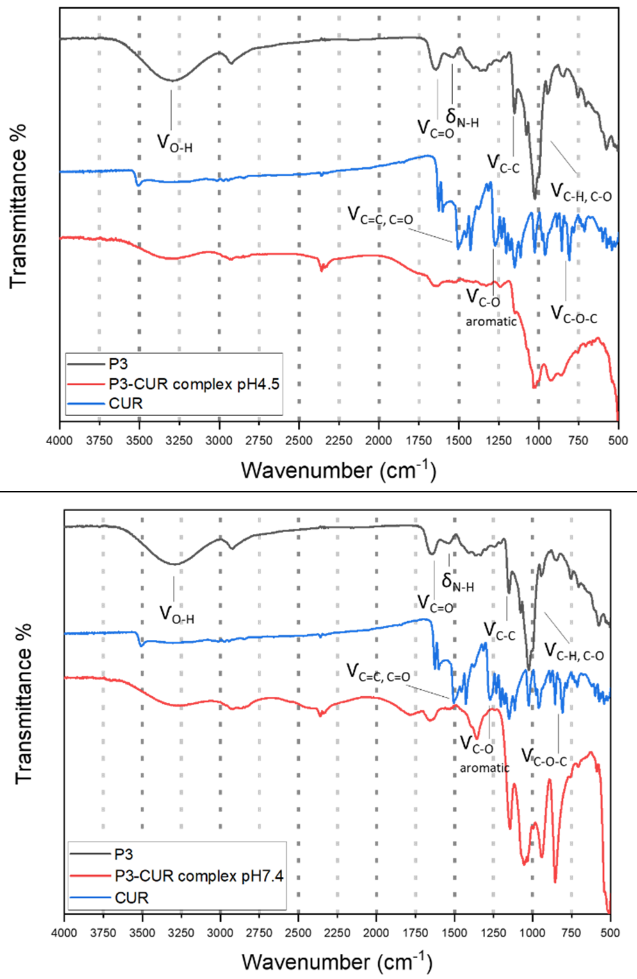

Figure S10. FT-IR/ATR spectra of P3-curcumin complexes at pH 4.5 and 7.4.

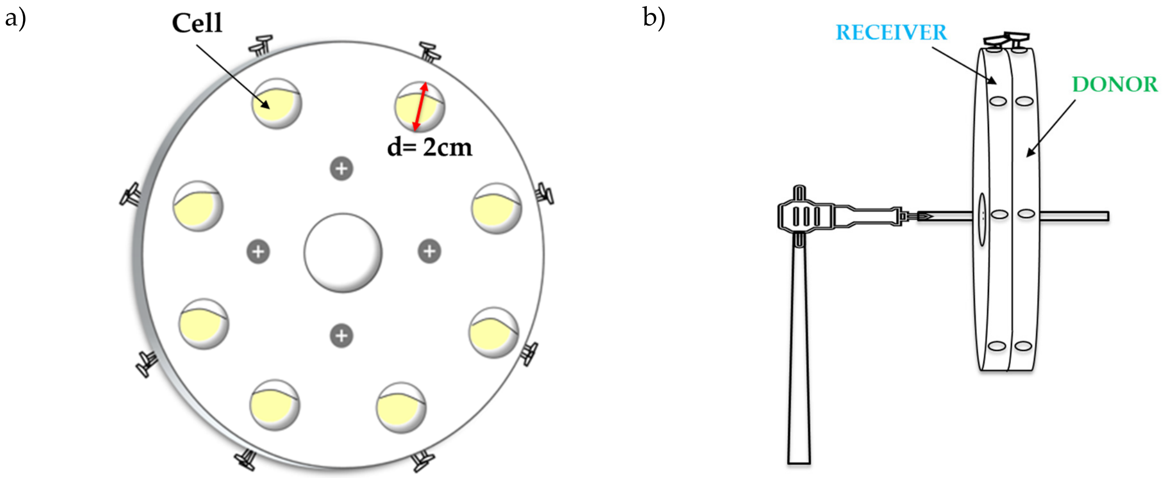

Figure S11. a) front and b) side view of custom-made multi-compartment rotating apparatus for *in vitro* release studies.

Calibration Curves

The HPLC and UV-Vis were calibrated with curcumin standard solutions over the concentration range of 2 to 20 ppm. A linear calibration curve was obtained over the concentration range.

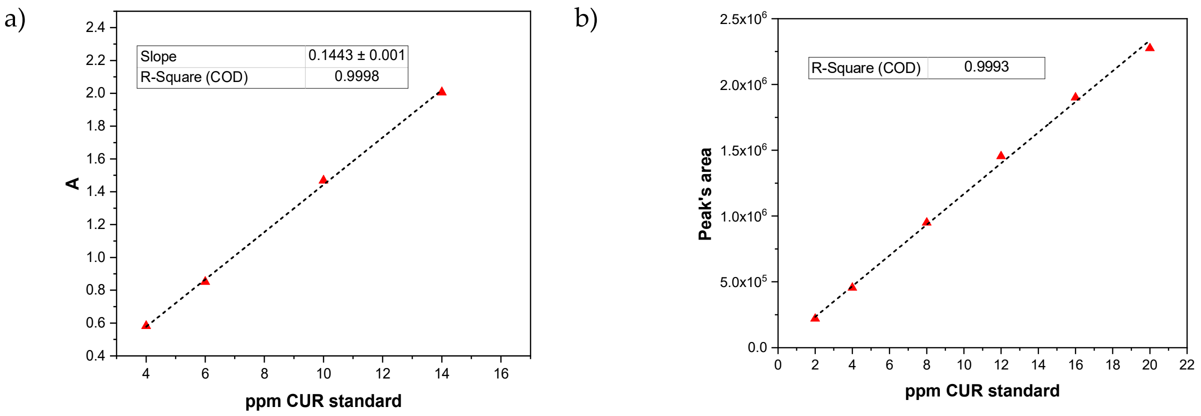

Figure S12. Calibration curve over the concentration range: a) 4 to 14 ppm for UV-vis analysis; b) 2 to 20 ppm for HPLC analysis.

Kinetic Studies

To study the mechanism of curcumin release from P3/P5-complexes, five mathematical models were taken in consideration: zero-order (F=kt), first-order (ln(1-F)= -kt), Higuchi´s model ($F=kt^{1/2}$), Hixon-Crowell´s model ($1-(1-F)^{1/3}$= kt) and Korsmeyer-Peppas model, or "the power law", ($F=kt^n$). The squared correlation coefficients (R^2) and slope (rate constant, k) obtained after linear regression by mathematical models considering drug release fraction (F) and time (t), as respectively y and x variables, are listed in Table S1.

P3-curcumin complex pH 4.5

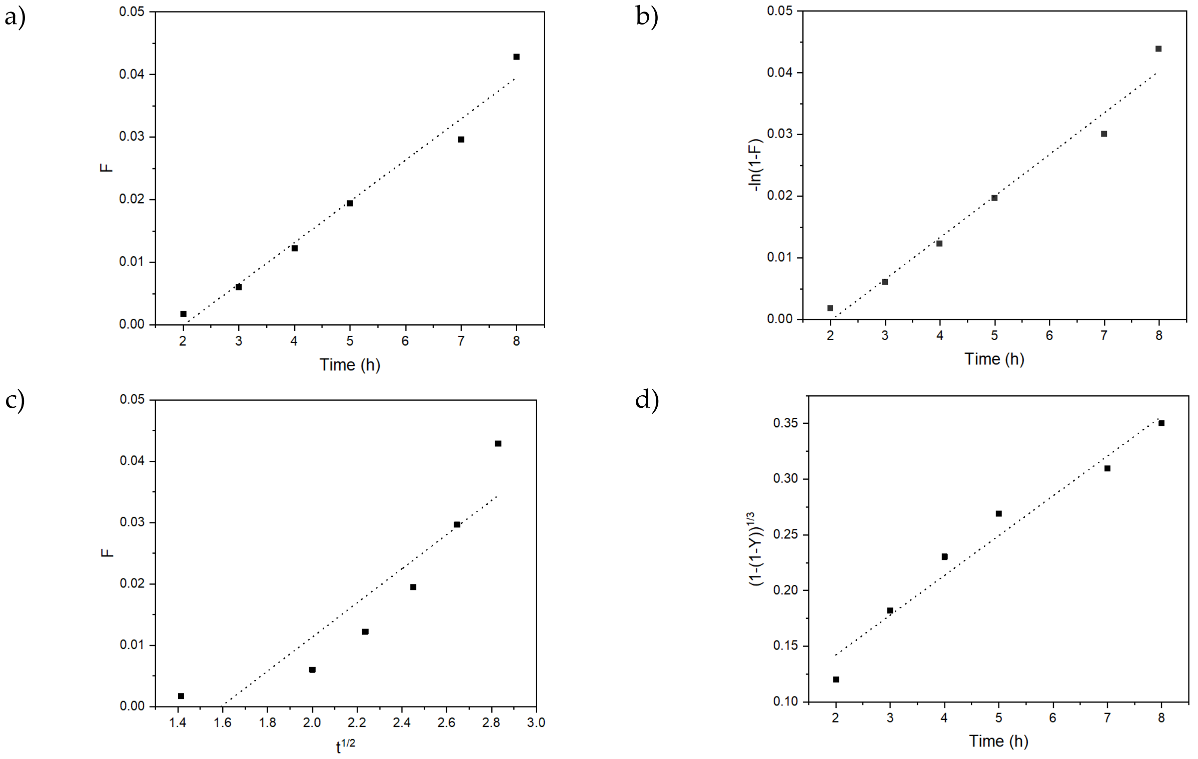

e)

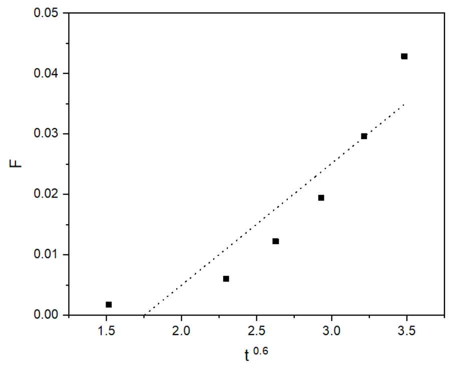

P3-curcumin complex pH 7.4

a)

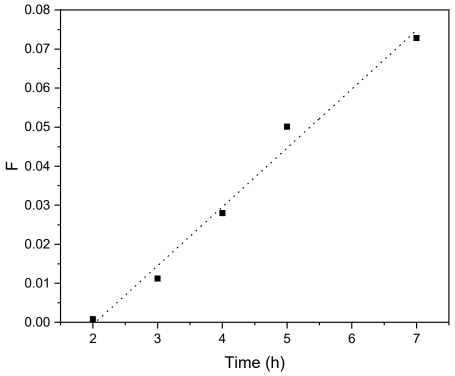

b)

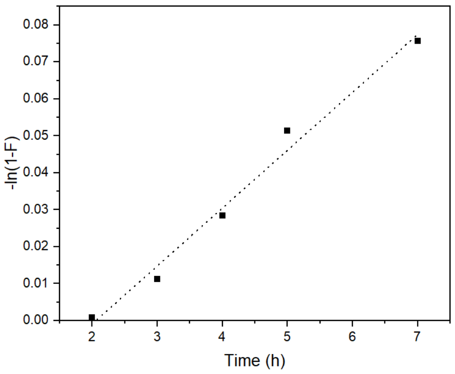

c)

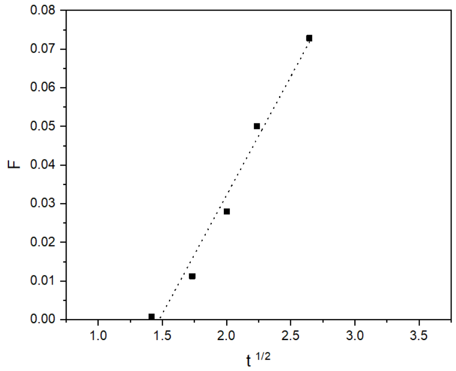

d)

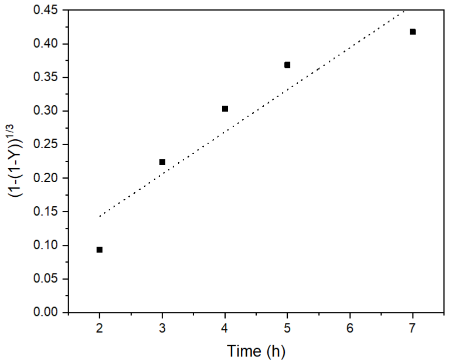

e)

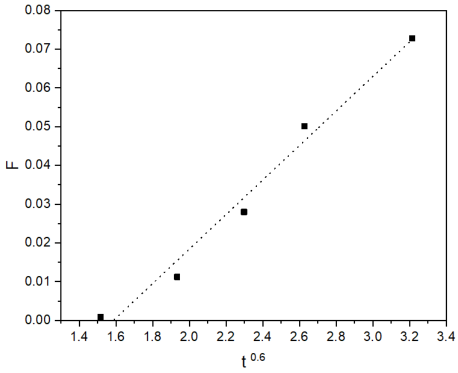

P5-curcumin complex pH 4.5

a)

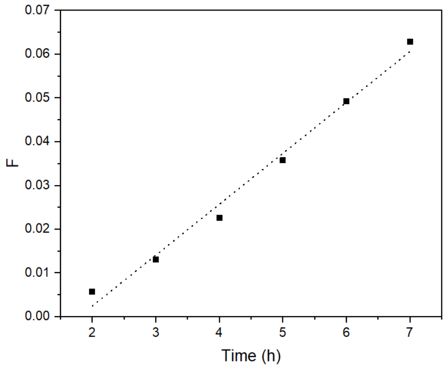

b)

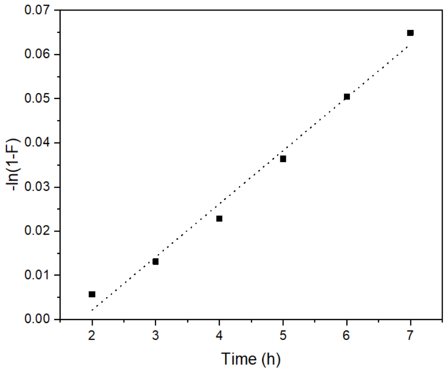

c)

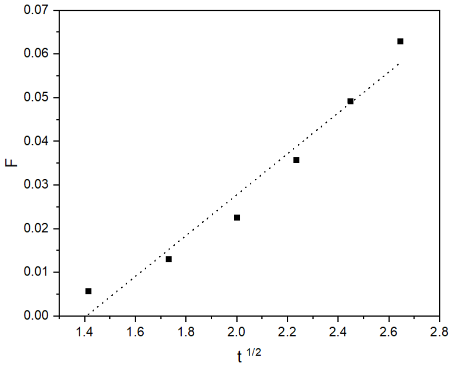

d)

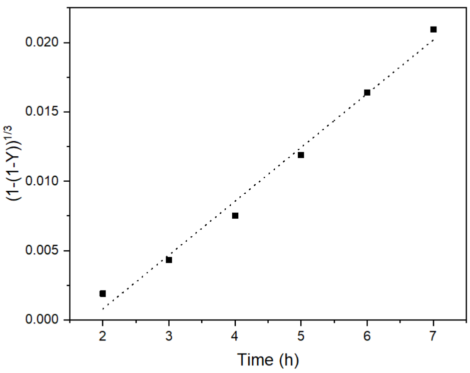

e)

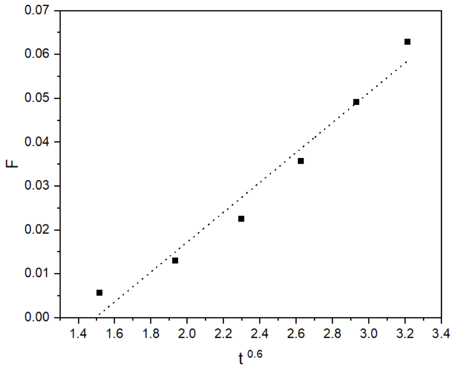

P5-curcumin complex pH 7.4

a)

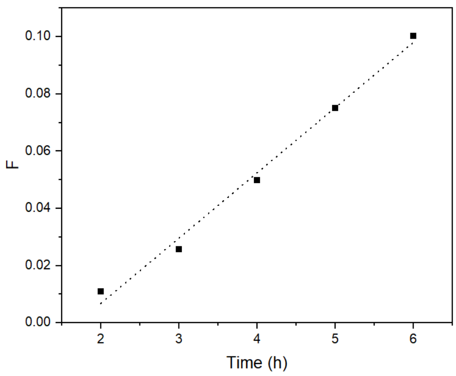

b)

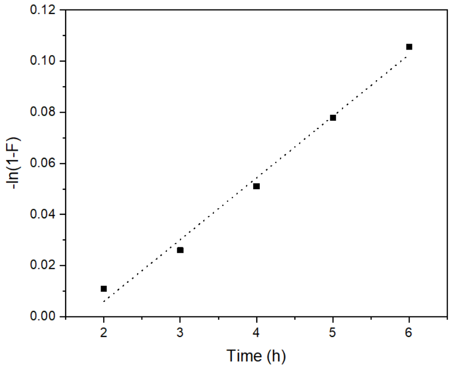

c)

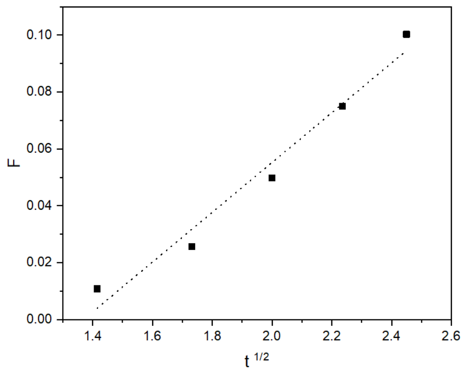

d)

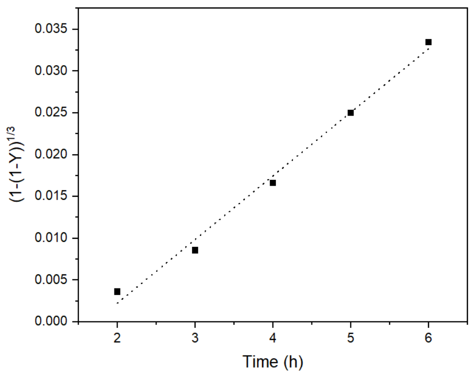

e)

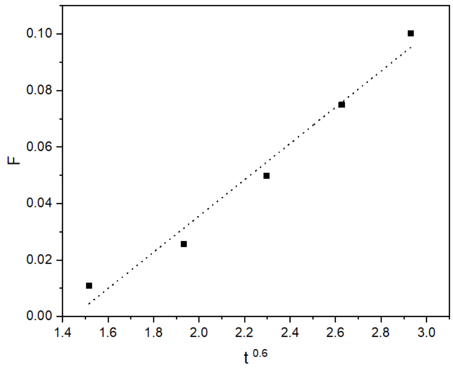

Figure S13. Linear regression by mathematical models of P3/P5-curcumin complex release curves: **a)** zero-order (F=kt), **b)** first-order (ln(1-F)= -kt), **c)** Higuchi´s model ($F=kt^{1/2}$), **d)** Hixon-Crowell´s model ($1-(1-F)^{1/3}= kt$), **e)** Korsmeyer-Peppas model ($F=kt^n$).

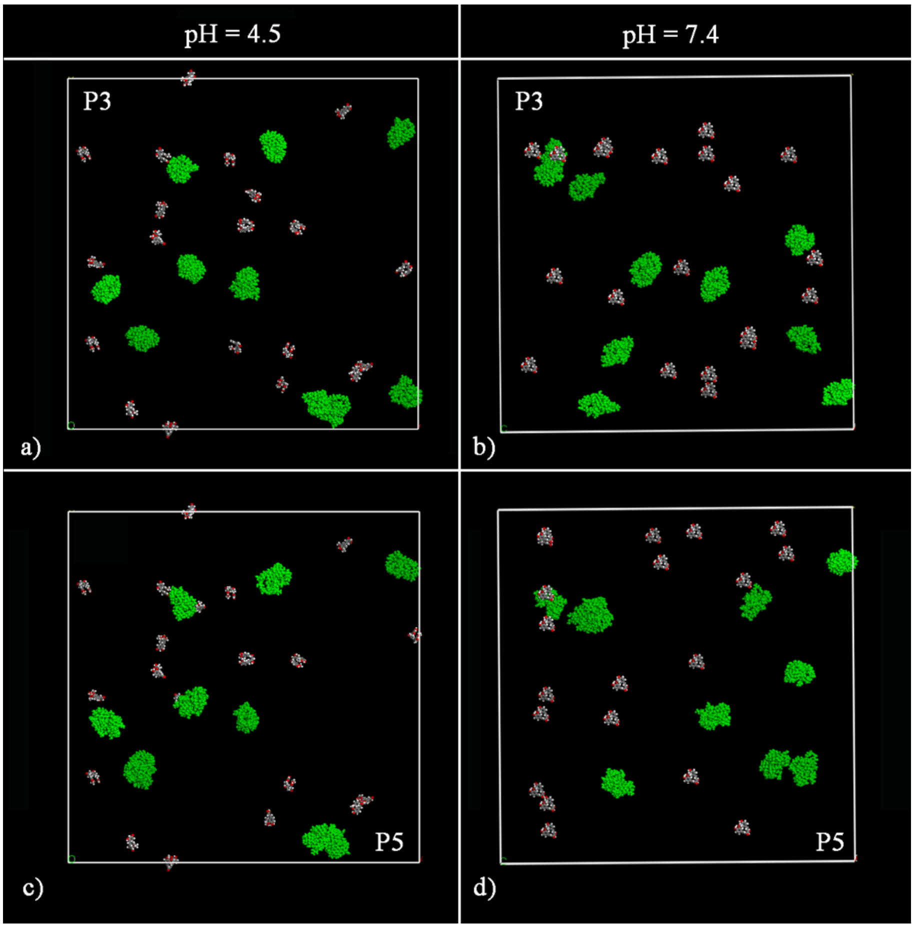

Figure S14. Initial non optimized geometries before the first energy minimization.
